# Molecular Dynamics Simulations of Crystal Nucleation near Interfaces in Incompatible Polymer Blends

**DOI:** 10.3390/polym13030347

**Published:** 2021-01-22

**Authors:** Wenlin Zhang, Lingyi Zou

**Affiliations:** Department of Chemistry, Dartmouth College, Hanover, NH 03755, USA; Lingyi.Zou.GR@dartmouth.edu

**Keywords:** crystallization, phase separation, interfaces, molecular dynamics simulations

## Abstract

We apply molecular dynamics (MD) simulations to investigate crystal nucleation in incompatible polymer blends under deep supercooling conditions. Simulations of isothermal nucleation are performed for phase-separated blends with different degrees of incompatibility. In weakly segregated blends, slow and incompatible chains in crystallizable polymer domains can significantly hinder the crystal nucleation and growth. When a crystallizable polymer is blended with a more mobile species in interfacial regions, enhanced molecular mobility leads to the fast growth of crystalline order. However, the incubation time remains the same as that in pure samples. By inducing anisotropic alignment near the interfaces of strongly segregated blends, phase separation also promotes crystalline order to grow near interfaces between different polymer domains.

## 1. Introduction

Phase separation and crystallization can occur sequentially or simultaneously during polymer processing, and in turn affect the final morphologies and material properties of polymer blends, such as recycled polyolefin mixtures, and electron–donor/acceptor conjugated polymers [1,2,3,4,5]. Over the past few decades, tremendous theoretical and experimental efforts have been made to understand the two transitions separately [6,7,8,9,10,11,12]. A fundamental understanding of the coupling between phase separation and crystallization for incompatible semicrystalline polymers, however, is still mostly lacking.

Using polarized optical microscopy (POM) and differential scanning calorimetry (DSC), previous works demonstrated that spinodal decomposition could accelerate crystallization in phase-separated polyolefin blends [3,13,14,15,16,17,18]. Crystallization from a phase separated blend is faster than that in a pure sample and crystalline spherulites prefer to form near the interfaces between polymer domains. To rationalize the experimental observations, Mitra and Muthukumar proposed a phenomenological model of heterogeneous nucleation, in which the domains of the incompatible polymer act as nucleation agents [19]. Thus, the free energy barrier for nucleation is lower for the crystallizable polymer segments near the interfaces, and the overall nucleation rate depends on the total interfacial area in the blend.

The accelerated nucleation may also arise from the interface-induced orientational order of polymer segments. Instead of nucleating directly from isotropic melts, polymers may nucleate crystalline order from a partially orientated precursor state. Han and coworkers proposed that the inter-diffusion of polymer segments across the interfaces during the spinodal decomposition could promote chains to align perpendicular to the interfaces [14,15,16]. However, the reptative inter-diffusion may not be sufficient to impose strong alignment to polymers at the monomer or the Kuhn segment level, which is critical to accelerating crystal nucleation [20,21,22]. Nevertheless, highly incompatible semiflexible polymers tend to align parallel to interfaces, creating alignment layers of a thickness about a Kuhn length [23,24,25]. The interface-induced alignment may help polymer segments nucleate crystalline order. For example, previous authors demonstrated using coarse-grained simulations that polymer chains tend to nucleate near flat and impenetrable surfaces with crystalline stems that align parallel to the walls [26].

To better understand the coupling between phase separation and crystallization, we apply molecular dynamics (MD) simulations to investigate the crystal nucleation in inhomogeneous polymers. Previous authors have used MD simulations to study the kinetics and the precursors of the quiescent [27,28,29,30,31,32,33] and flow-induced [20,21,22,31,34,35] nucleation and crystallization in homopolymer melts. Computational studies of crystal nucleation in inhomogeneous polymer blends, however, are still lacking.

We perform simulations of isothermal nucleation for phase-separated blends of polyethylene (PE) and isotactic polypropylene (iPP) oligomers. PE and iPP are arguably the most important commercial thermal plastic polymers, used in numerous applications, ranging from packaging to healthcare products. Studying the effects of phase separation on the crystallization of PE/iPP blends is essential for designing better recycling strategies for mixed polyolefin wastes [36]. Because PE and iPP oligomers are only mildly incompatible, the phase-separation-induced interfacial ordering is negligible. In order to study the effect of interfacial ordering on crystal nucleation, we create blends of artificial PE${}_{a}$ and iPP${}_{a}$ with an enhanced incompatibility by tuning the inter-species interactions between PE and iPP. The enhanced incompatibility leads to polymer alignment near the interfaces.

For weakly segregated PE/iPP blends, PE crystal nucleation tends to occur in the bulk region where the PE concentration is high. The iPP impurities, which are less mobile than PE, significantly impede the nucleation kinetics, resulting in a longer incubation time and a slower growth of crystalline nuclei than those in pure PE samples. When PE oligomers are phase-separated from a more mobile polymer, the impurity in the interfacial regions promotes the growth of crystalline order. In strongly segregated artificial PE${}_{a}$/iPP${}_{a}$ blends, although the nucleation kinetics of $PE_{a}$ is similar to that in the bulk melt, the crystalline order prefers to grow in the alignment layers near the interfaces during the late stage of nucleation.

## 2. Methods

We perform MD simulations of incompatible polymer chains using the GROMACS 2019.2 package [37]. In our simulations, the PE and iPP oligomers are modeled using the united-atom TraPPE force field [38,39]. The reduced molecular details in the TraPPE model help keep the cost for simulating polymer crystallization manageable. Previous authors showed that the TraPPE model could yield melt properties and melting temperatures $T_{m}$ that were consistent with experiments for n-alkane and PE [29,30,40,41]. The TraPPE model has also been used to study the flow-induced nucleation of iPP [34]. In the current work, we only analyze the crystal nucleation of PE because iPP nucleates much slower than PE under quiescent conditions.

The non-bonded interactions for PE and iPP are modeled using a Lennard–Jones potential:

(1) $$U_{nb}\left(r_{i,j}\right)=4\epsilon_{i,j}\left[{\left(\frac{\sigma_{i,j}}{r_{i,j}}\right)}^{12}-{\left(\frac{\sigma_{i,j}}{r_{i,j}}\right)}^{6}\right]$$

where the indices i,j represent different atoms (which in general can be of different types). In our simulations, the non-bonded interactions are truncated and shifted to zero at a cut-off distance of 0.14 nm.

The bonded interactions in our simulations include the harmonic bond stretching and bending potentials, and the dihedral potentials for the torsional rotation of polymer backbones. The bond stretching potential is

(2) $$U_{b}\left(l\right)=\frac{k}{2}{\left(l-l_{0}\right)}^{2}$$

in which the spring constant *k* is 502,416.0 kJ/mol/nm${}^{2}$ and the equilibrium length of carbon-carbon bond $l_{0}$ is 0.154 nm. The spring constant *k* is not included in the original TraPPE model [38,39], which was designed for Monte Carlo simulations of molecules with fixed bond lengths. The spring constant *k* here is borrowed from the CHARMM force field model [42]. In our simulations, we use linear constraint solver (LINCS) algorithm [43] to constrain the bond length. Although LINCS and a large time step (4 fs) work well for simulating pure iPP and PE, they can generate artificial density fluctuations in the binary blends of PE/iPP. We will discuss and warn the readers about this artifact later.

The harmonic bond bending potential is

(3) $$U_{\theta }\left(\theta \right)=\frac{k_{\theta }}{2}{\left(\theta -\theta_{0}\right)}^{2}$$

where $k_{\theta }$ is 519.7 kJ/mol/rad${}^{2}$ and the equilibrium bond angle $\theta_{0}$ slightly depends on the bond types. Nevertheless, all the equilibrium bond angles are about 114${}^{\circ }$ in iPP and PE.

The potential for rotating the backbone dihedral angle ϕ is written in the form of a Ryckaert–Bellemans function:

(4) $$U_{\phi }\left(\phi \right)=\sum_{n=0}^{5}C_{n}{\left(\cos\left(\phi \right)\right)}^{n}$$

in which the coefficients $C_{n}$ depend on dihedral angle types and can be found in the original TraPPE paper [38,39]. Together with the bond angles, the dihedral potential governs the persistence length of polymers [44]. The value of persistence length is critical to surface induced nematic order in polymer samples [23,24].

To prepare phase-separated blends of PE and iPP oligomers, we first separately simulate PE and iPP oligomers of 50 repeating units between impenetrable walls parallel to the xy-plane. The initial configurations of PE and iPP melts are composed of 324 and 216 loosely packed chains, respectively. The PE and iPP oligomers are not entangled in our simulations because the chain lengths are shorter than the critical entanglement lengths [45]. The unentangled polymer chains can relax their configurations quickly in the melt state. The nucleation time of the unentangled PE oligomers is also relatively short to keep the overall computational cost manageable.

The interactions between polymer atoms and the wall is governed by a LJ type of potential:

(5) $$U_{wall}\left(z\right)=4\epsilon_{wall}\left[{\left(\frac{\sigma_{wall}}{z}\right)}^{12}-{\left(\frac{\sigma_{wall}}{z}\right)}^{6}\right]$$

in which *z* is the distance between a polymer atoms and a wall in the *z*-direction, $\epsilon_{wall}$ is $9.13\times 10^{-4}$ kJ/mol and $\sigma_{wall}$ is 0.1 nm. We use walls to confine polymer chains in the simulation box so that no segment resides outside the simulation box in the *z*-direction.

**Figure 1:**
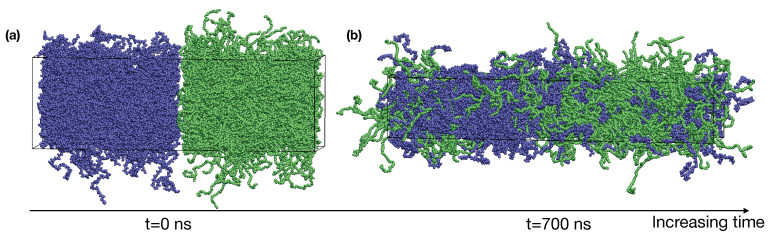
(**a**) A snapshot of the initial configuration of PE/iPP with sharp interfaces; (**b**) an equilibrated PE/iPP blend at 400 K. iPP (blue), PE (green).

We dynamically shrink the confined blends of PE and iPP with constant deformation rates in *x*, *y*, and *z* directions at 400 K until the box dimensions become 9×9×14 nm${}^{3}$, which corresponds to a density of about 0.7 g/cm${}^{3}$. The deformation is performed using the deform option of GROMACS. By placing the PE and iPP slabs next to each other in the *z*-direction, we create the initial configuration for a roughly 50:50 (by volume) blend of PE and iPP with two sharp interfaces (Figure 1a).

To equilibrate the binary blend of PE and iPP oligomers, we perform NPT simulations at 400 K for 900 ns. The pressure in our NPT simulations is 1 bar. A time step of 2 fs is used in our simulations. We use semi-isotropic pressure coupling (the pressure coupling is only isotropic in the $\hat{x}$ and $\hat{y}$ directions) so that the box dimensions can vary independently in the $\hat{x}/\hat{y}$ direction and the $\hat{z}$ direction. During the NPT simulations, the interfaces broaden until the equilibrium interfacial compositional profiles are formed. The simulation box also undergoes a shape change to relax the excess stress in the molten blend and finally fluctuates about the size of 7×7×39 nm${}^{3}$ (Figure 1b). The box dimension in *x* and *y* is greater than the radius of gyration of PE and iPP oligomers, of about 1.7 nm. Thus, polymers in our simulations do not interact with themselves across the periodic boundaries. Our simulation time is much longer than the self-diffusion time τ for PE and iPP oligomers at 400 K, which is 56 ns and 110 ns, respectively. The value of τ is calculated from our blend simulations as $R_{g}^{2}/D_{com}$, in which $R_{g}$ is the radius of gyration and $D_{com}$ is the diffusion constant of the polymer center-of-mass. The compositional profiles of PE and iPP are also stable over the last 300 ns in our simulations. Thus, the binary blend of PE and iPP is equilibrated.

**Figure 2:**
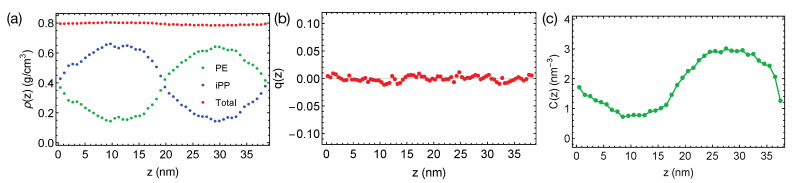
(**a**) Density profiles; (**b**) distribution of nematic order; (**c**) concentration of all-trans PE atoms in the binary blend of PE/iPP oligomers at 400 K.

The rather short PE and iPP oligomers are only mildly incompatible, giving rise to the broad interfaces in simulations at 400 K (Figure 2a). The bulk regions of PE and iPP domains are narrow and include noticeable amounts (about 25% by volume) of chains from the opposite phases. By fitting the bulk volume fraction of PE to the Flory–Huggins theory, we estimate the Flory–Huggins χ, which quantify the effective repulsion between different polymers, to be about 0.047 (per PE monomer), indicating the system is close to the phase boundary. A slightly increased simulation temperature of 420 K leads to mixing PE and iPP oligomers.

Together with the small χ, the flexible backbones of PE and iPP (the persistence length $N_{p}$ for PE and iPP is about three repeating units) lead to the negligible interface-induced orientational ordering. For a weakly segregated symmetric blend ($N_{p}\chi \ll 1$), the maximum surface-induced nematic order is $-N_{p}\chi /12$, where the negative sign indicates polymer segments align parallel to the interface [23]. We expect PE and iPP segments to exhibit a nearly zero nematic order near the interfaces. We indeed observe that PE and iPP are disordered everywhere in the simulation box, exhibiting a negligible nematic order $q\left(z\right)=\langle P_{2}\left(t\left(z\right)\cdot \hat{z}\right)\rangle$ across the simulation box in the $\hat{z}$ direction, where *t* is a unit backbone tangent vector and $P_{2}$ is the second order Legendre Polynomial (Figure 2b).

We do not observe any conformational order induced by the interfaces. By conformational order, we mean all-trans conformation for PE oligomers. Because PE crystals are composed of all-trans PE chains, we expect the all-trans segments to be important for PE crystallization. Previous authors have demonstrated that the conformationally ordered polymer segments are precursors to flow-induced crystallization [46]. To show the distribution of conformational order in the binary blend, we plot the concentration of all-trans backbone atoms for PE oligomers in the blend (Figure 2c). An all-trans segment is defined to be three monomers ($CH_{2}CH_{2}$) with six successive trans dihedral angles ($-60^{\circ }<\phi <60^{\circ }$). The concentration of all-trans backbone atom varies smoothly from the iPP domain to the PE domain, with a profile similar to the shape of the density profile of PE.

**Figure 3:**
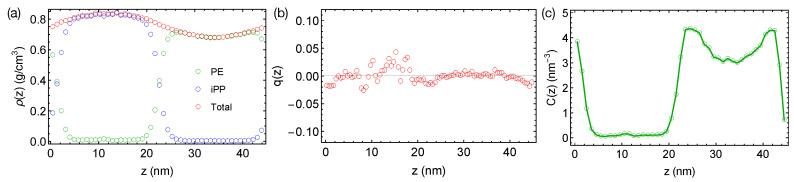
(**a**) Density profiles; (**b**) distribution of nematic order; (**c**) concentration of all-trans PE atoms in the binary blend of PE/iPP oligomers simulated using LINCS and a time step of 4 fs at 450 K.

Now, we want to discuss the artifact caused by a larger time step and LINCS in the PE/iPP simulations. When a time step of 4 fs is used with LINCS in simulations, sharp interfaces between PE and iPP are stable over 400 ns even at an elevated temperature of 450 K (Figure 3a). The sharp interfaces indicate an over-predicted χ, which is still not large enough to induced nematic order to the polymer segments (Figure 3b). We also observe an artificial and sinusoidal density fluctuation across the simulation box, which leads to a high-density region of the iPP phase and a low-density region of the PE phase. The densities of PE and iPP oligomers in the bulk regions are different from the densities we observed in pure melts of PE and iPP oligomers at 450 K, which are all about 0.75 g/cm${}^{3}$. The density of PE and the concentration of all-trans segments are also higher near the interfaces than in the bulk PE region (Figure 3c). The artificial incompatibility, density fluctuations, and conformational ordering are resolved when the time step is reduced to 2 fs or when LINCS is turned off. Because the TraPPE model uses rather common interaction types, we speculate that the artifact is not associated with the force field model. We suggest readers carefully choose the time step in simulations of phase-separated polymer blends when LINCS is used.

To study the crystal nucleation in strongly segregated polymer blends, we adjust the Flory–Huggins χ between PE and iPP by reducing the attraction between the junction atoms (CH) of iPP and PE atoms. Indeed, the incompatibility between PE and iPP increases with decreasing temperature or increasing molecular weight. Still, low temperatures and high molecular weights lead to slow polymer relaxations. To keep the computational cost tractable, we increase χ for PE and iPP by reducing the attractive part in the non-bonded interactions between the junction atoms of iPP and PE atoms:

(6) $$U_{nb}^{\lambda }\left(r\right)=\frac{4\epsilon }{\lambda^{12}}\left[{\left(\frac{\sigma \lambda }{r}\right)}^{12}-{\left(\frac{\sigma \lambda }{r}\right)}^{6}\right]$$

The repulsion in the non-bonded interaction remains unchanged and the attraction decreases with increasing λ. Thus, the Flory–Huggins χ increases with increasing λ. We fix the repulsion to keep the size of the coarse-grained united atoms unchanged. Because we only adjust the inter-species interactions, the crystal melting temperatures of PE${}_{a}$ and PE oligomers are the same.

**Figure 4:**
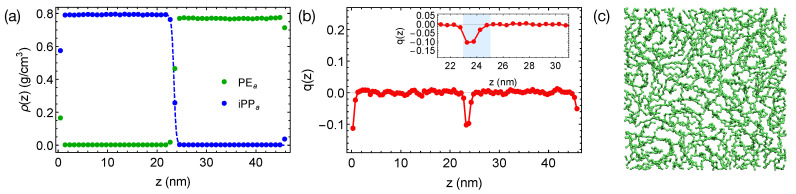
(**a**) Density profiles; (**b**) distribution of nematic order in the PE${}_{a}$/iPP${}_{a}$ blend. Dashed curve in (**a**) is a fit to HT theory [47]. Blue region in the inset of (**b**) demonstrates an orientationally ordered region near a planar interface in the PE${}_{a}$ domain; (**c**) a snapshot of chain segments in the orientationally ordered region.

We equilibrate the artificial blends of PE${}_{a}$ and iPP${}_{a}$ with λ=1.2 at 420 K for 400 ns, much longer than the self-diffusion time τ of PE chains, which is 21 ns at 420 K. By reducing the inter-species attraction, we increase the incompatibility between PE and iPP, which leads to sharper interfaces in simulations (Figure 4a). By fitting the interfacial width of 0.47 nm to the Helfand and Tagami (HT) theory [47], we estimate χ of the “artificial” PE${}_{a}$/iPP${}_{a}$ blend to be about 0.32, much greater than χ of natural PE/iPP. The strongly segregated blend also exhibits a weak interface-induced nematic order (see the inset of Figure 4b). Both of the PE${}_{a}$ and iPP${}_{a}$ segments tend to align parallel to the interface. For example, an alignment layer of a thickness about 2 nm can be observed in the PE${}_{a}$ domain (blue shade in Figure 4b). In the alignment layers, however, polymer segments are rather disordered in the transverse directions (Figure 4c).

To investigate the isothermal crystal nucleation for PE and PE${}_{a}$, we extract eight different melt configurations from the equilibrated trajectory for each molten blend of PE/iPP and PE${}_{a}$/iPP${}_{a}$. The initial melt configurations are quenched to 300 K to trigger isothermal crystallization of PE and PE${}_{a}$. The crystallization temperature here is much lower than the crystal melting temperature of PE and PE${}_{a}$ oligomers of 100 carbon atoms, about 389 K [48]. We use the rather deep supercooling condition to access fast crystal nucleation.

To reference the nucleation behaviors in the phase-separated blends, we also perform simulations to obtain the nucleation rate for pure PE oligomers. A pure melt of 324 PE oligomers of 100 backbone carbon atoms is equilibrated at 420 K and 1 bar for 400 ns, from which eight configurations are extracted. The eight melt configurations are subsequently quenched to 300 K to crystallize. By comparing the nucleation rates of PE and PE${}_{a}$ in binary blends with that in pure samples, we show the effects of phase separation and interfaces on the crystal nucleation kinetics.

## 3. Results

In this work, we only study the crystal nucleation of PE and PE${}_{a}$ because iPP and iPP${}_{a}$ are too slow to nucleate under quiescent conditions in simulations. The crystalline order of PE atom is identified using a local bond order parameter $q_{6}q_{6}^{*}$, which quantifies the correlation among the six-order Steinhardt order parameters of a given atom and its neighboring atoms (within a cut-off distance of 0.54 nm) [30,49,50]. A local bond order parameter greater than 2.2 can distinguish crystalline atoms of united-atom PE (hexagonal packing) from isotropic atoms.

**Figure 5:**
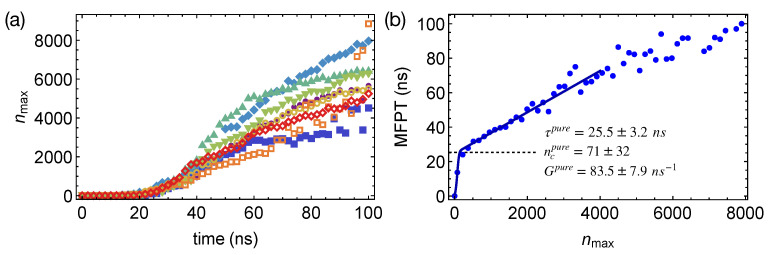
(**a**) Growth of the largest nucleus in eight simulations of isothermal nucleation of pure PE oligomers; (**b**) mean-first-passage time (MFPT) for the largest crystalline nucleus reaching size $n_{max}$ in pure PE oligomers, fitting to the classical nucleation theory (curve); incubation time $\tau^{pure}$ marked by a dashed line.

To quantify the nucleation kinetics, we define crystal nuclei by grouping neighboring crystalline atoms within a cut-off distance of 0.54 nm. By tracking the spatial and temporal evolution of crystalline order in the PE or the artificial PE${}_{a}$ domains, we obtain the preferred locations of crystalline nuclei in phase-separated polymer blends. We also compute the mean-first-passage time (MFPT) for the formation of the largest crystalline nucleus by averaging over the different simulation trajectories (Figure 5a). By fitting the classical nucleation theory [27] to the MFPT data:

(7) $$MFPT\left(n_{max}\right)=\frac{1}{2}\tau \left[1+erf\left(Z\pi^{1/2}\left(n_{max}-n_{c}\right)\right)\right]+\frac{1}{2G}\left(n_{max}-n_{c}\right)\left[1+erf\left(C\left(n_{max}-n_{c}\right)\right)\right]$$

where *Z* is the Zeldovich factor and *C* is a sufficiently large positive number, we can extract the incubation time τ, the critical nucleus size $n_{c}$, and the growth rate *G* of crystalline nucleus to quantify the crystal nucleation behaviors in different polymer samples. For pure PE oligomer samples at 300 K, we obtain MFPT for the largest crystalline nucleus to reach the size of $n_{max}$ carbon atoms (Figure 5b), from which $\tau^{pure}$, $n_{c}^{pure}$, and $G^{pure}$ are estimated to be 25.5±3.2 ns, 71±32 carbon atoms, and 83.5±7.9 carbon atoms per ns, respectively (summarized in Table 1). The error bars represent the 95% confidence intervals of the fitting parameters. We notice that the critical nucleus of PE is much smaller than the critical nucleus of molecules with roughly isotropic shapes, such as LJ liquids and colloidal particles [51,52]. The rather small critical nucleus size of PE may result from the deep supercooling and the multi-stage crystal nucleation of polymers. Unlike molecules with isotropic shapes, polymers are chain-like, which can form a metastable nematic phase below the crystallization temperature [30]. By crystallizing from the partially ordered nematic precursors, in which chain segments align uniaxially, the nucleation barrier and the critical nucleus size may be reduced for polymers.

**Figure 6:**
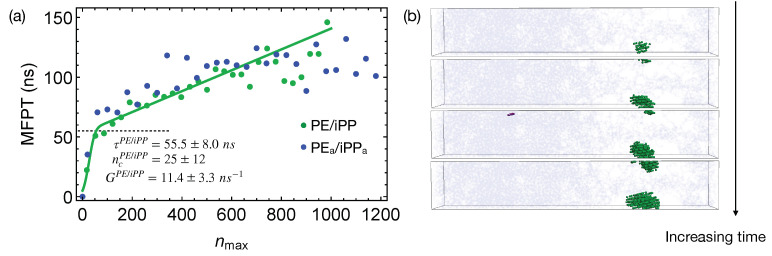
(**a**) MFPT for the largest crystalline nucleus to reach size $n_{max}$ in weakly segregated blends of PE/iPP oligomers and incompletely segregated blends of PE${}_{a}$/iPP${}_{a}$; fitting PE/iPP data to classical nucleation theory (curve). Incubation time $\tau^{PE/iPP}$ marked by a dashed line; (**b**) snapshots of the PE/iPP oligomer blends during isothermal nucleation. iPP atoms (light blue). Crystalline PE atoms in different nuclei (blue and purple). Amorphous PE not shown for clarity.

Quenching the weakly segregated blends of natural PE and iPP (Figure 6) to 300 K leads to slow crystal nucleation. By fitting the MFPT for the formation of the largest crystalline nucleus, we obtain the incubation time $\tau^{PE/iPP}$ to be 55.5±8.0 ns, longer than the incubation time $\tau^{pure}$ for crystallizing pure PE oligomers. The growth rate $G^{PE/iPP}$ is 11.4±3.3 ns${}^{-1}$, much slower than the growth rate of PE nuclei in the pure samples (83.5±7.9 ns${}^{-1}$). The nucleation kinetics is significantly impeded by iPP in the PE domain. In fact, the crystalline atoms emerge near the center of the PE domain, where the PE concentration is high (Figure 6b). iPP segments need to be expelled from the growing PE nuclei because they cannot be included in PE crystals. This is expected because, even for lightly methyl-branched PE, segments with methyl side groups are excluded from the growing crystalline nuclei during isothermal nucleation [49]. The slow expulsion of iPP from PE nuclei, resulting from the low mobility of iPP segments, can hinder the crystal nucleation and growth.

The critical nucleus size $n_{c}^{PE/iPP}$ in the weakly segregated blends of PE and iPP oligomers is slightly smaller than the critical nucleus size $n_{c}^{pure}$ in pure PE oligomers (Figure 6a). The smaller critical nucleus size suggests that the free energy barrier ΔG for PE oligomers to nucleate in the weakly segregated blend is lower than ΔG of pure PE oligomers. Small amounts of iPP oligomers in the PE domain may slightly lower the nucleation barrier. Nevertheless, the slow iPP segments still hinder the overall nucleation kinetics for PE chains. At a higher crystallization temperature, a reduced nucleation barrier may overcome the hindrance imposed by the slow iPP dynamics, and in turn accelerates nucleation kinetics of PE. To quantify and confirm the effect of iPP oligomers on the nucleation barrier of PE, calculation of the surface free energy γ of PE crystal in pure melt and inhomogeneous melt is necessary. Together with the free energy difference between crystalline and molten PE, γ governs the nucleation barrier. The calculation of the surface free energy, however, is beyond the scope of our current work.

The incompatibility between different polymers only affects nucleation kinetics under deep supercooling conditions by impacting the phase separated morphologies before crystallization. During isothermal nucleation, the effective repulsion between different polymers only weakly affects the nucleation kinetics. To show this, we perform simulations of nucleation in PE${}_{a}$/iPP${}_{a}$ using the weakly segregated melt of natural PE/iPP as the initial configurations. The weakly segregated melt configurations mimic the intermediate stage of the spinodal decomposition where the concentrations in the PE${}_{a}$ and iPP${}_{a}$ domains are still different from the equilibrium values. When the incompletely separated PE${}_{a}$/iPP${}_{a}$ are quenched to 300 K, the growth of the largest crystalline nucleus in the PE${}_{a}$ domain is similar to the growth of crystalline PE nucleus in PE/iPP (Figure 6a).

**Figure 7:**
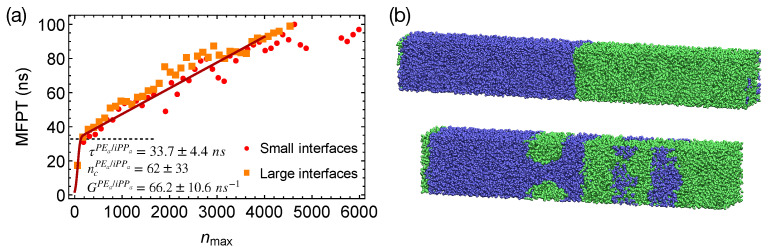
(**a**) MFPT for the largest crystalline nucleus to reach size $n_{max}$ in strongly segregated blends of PE${}_{a}$/iPP${}_{a}$ oligomers. Fit to classical nucleation theory (red curve); (**b**) snapshots of PE${}_{a}$/iPP${}_{a}$ with small interfaces (**upper**) and large interfaces (**lower**) before crystallization. PE${}_{a}$ (green) and iPP${}_{a}$ (blue).

In strongly segregated blends of PE${}_{a}$ and iPP${}_{a}$, although polymer segments tend to align parallel to the interfaces, creating ordered layers, we do not observe accelerated nucleation in the PE${}_{a}$ domains. The incubation time for the formation of a critical nucleus $\tau^{PE_{a}/iPP_{a}}$ in the PE${}_{a}$/iPP${}_{a}$ blends is 33.7±4.4 ns, comparable with the incubation time in pure PE (Figure 7a). The critical nucleus size $n_{c}^{PE_{a}}$ is 62±33 carbons, similar to the critical nucleus size in pure PE melt.

To show that the interfaces in strongly segregated blends impose negligible effects on the formation of a critical nucleus, we equilibrate PE${}_{a}$/iPP${}_{a}$ from the configurations of the weakly segregated PE/iPP blends at 420 K. By doing so, small domains of PE${}_{a}$ and iPP${}_{a}$ form, which lead to a total interfacial area larger than that of the two planar interfaces in our previous PE${}_{a}$/iPP${}_{a}$ blends (Figure 7b). By quenching PE${}_{a}$/iPP${}_{a}$ with multiple domains to 300 K, we show that the MFPT for the formation of the largest crystalline nucleus is almost the same as the MFPT we obtained from the simulations of PE${}_{a}$/iPP${}_{a}$ with two planar interfaces (Figure 7a). The interfaces in strongly segregated blends impose negligible effects on the formation of a critical nucleus and the incubation time τ.

**Figure 8:**
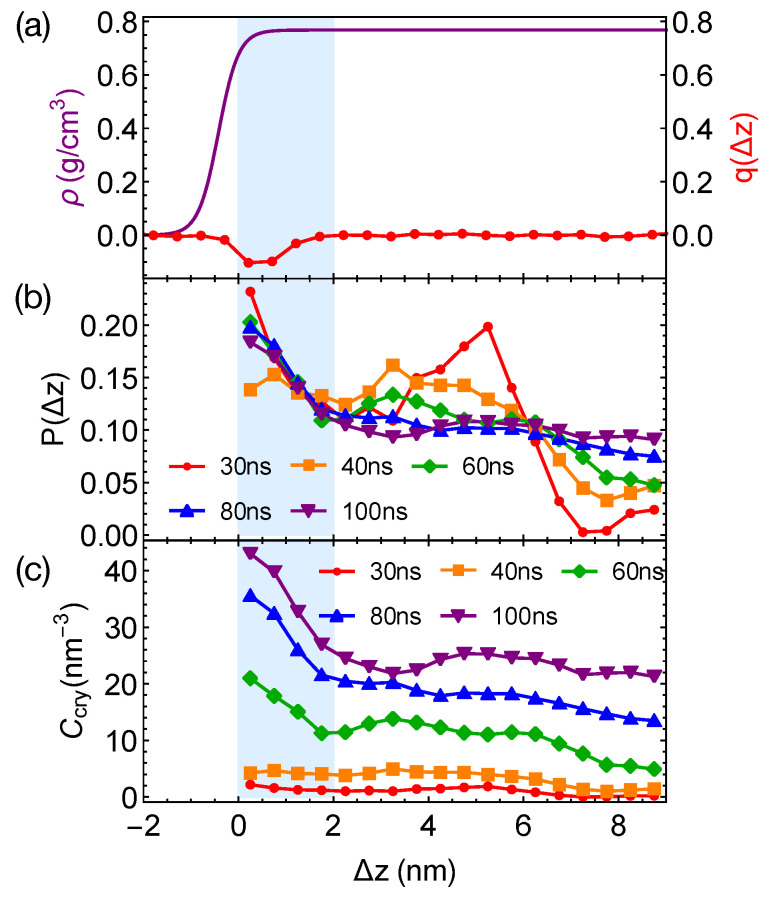
(**a**) Hyperbolic density profile of $PE_{a}$ by fitting data in Figure 4a and nematic order induced by interfaces; (**b**) probability distribution of crystalline atoms vs. distance Δz from the closest planar interfaces at different simulation time; (**c**) concentration of crystalline atoms vs. Δz at different simulation time. Data in (**b**,**c**) are averaged over eight trajectories. The blue region indicates the orientationally ordered layer in $PE_{a}$ domains.

**Figure A1:**
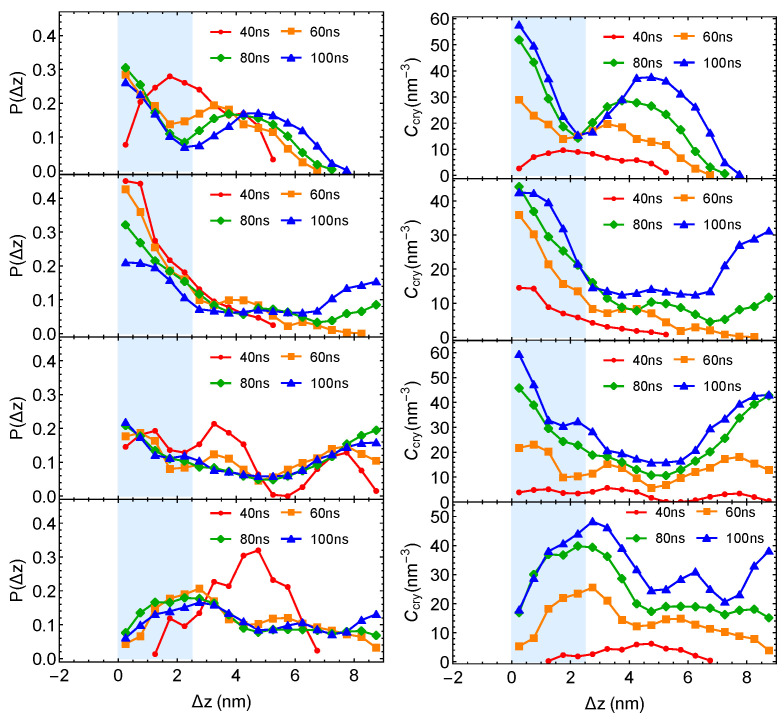
Crystalline atom distributions at different simulation time from four independent simulations of PE${}_{a}$/iPP${}_{a}$. (**Left column**): probability distribution of crystalline atoms. (**Right column**): concentration of crystalline atoms.

Although the formation of a critical nucleus is not accelerated by the interfaces and the interface-induced orientational order, crystalline atoms prefer to form near the interfaces in the strongly segregated blends, especially during the late stage of crystal nucleation. To show this, we plot the distributions of crystalline atoms at different times after the melts with planar interfaces are quenched to 300 K (Figure 8b,c). The distributions of crystalline atoms are averaged over different simulation trajectories. The evolution of the crystalline atom distribution for independent simulation trajectories can be found in Appendix A (Figure A1). Before nucleation (30 ns after quenching), crystalline atoms can form in both the alignment layers and the bulk regions. The deep supercooling is sufficient to induce the instantaneous formation of nematic precursors in the bulk region, in which crystalline order nucleates rapidly [30]. As a consequence, the interface-induced nematic order cannot accelerate crystal nucleation by promoting the formation of nematic precursors, which can readily form in the bulk regions. The weak effect of the interfaces on nucleation is similar to the mild effect of flow-induced nematic order on crystal nucleation at low crystallization temperatures [22]. Nevertheless, the interface-induced nematic order may accelerate crystal nucleation at high crystallization temperatures where the instantaneous formation of nematic precursors is not available. The growth of crystalline order, however, is faster in the orientationally ordered layers near the interfaces. Both the probability and the number density of crystalline atoms $C_{cry}$ remain high within the range of 2 nm from the opposite phase over the simulation time. The probability for finding crystalline atom in the alignment layer also starts to outgrow the probability for crystalline atom in the bulk region at 40 ns after quenching the polymer blends to 300 K.

## 4. Discussion

At the deep supercooling condition, we do not observe any accelerated crystal nucleation in blends of weakly segregated PE/iPP and strongly segregated PE${}_{a}$/iPP${}_{a}$ oligomers. In weakly segregated PE/iPP blends, PE oligomers even nucleate significantly slower than they do in pure samples. Our observations are different from the experimentally measured crystallization in polyolefin blends under weak supercooling conditions, where the phase separation accelerates crystallization and promotes the formation of crystallites near domain boundaries. Indeed, we observe that crystalline nuclei prefer to grow in the orientationally ordered layers near the interfaces in strongly segregated blends. Still, natural polyolefins may not be incompatible enough to induce interfacial alignment during the spinodal decomposition, and in turn promote the formation of crystallites near the interfaces.

The slow dynamics of iPP may lead to the absence of the phase-separation-accelerated nucleation and crystallization, which is observed in phase-separated blends, such as poly-(ethylene-co-hexene) (PEH) and poly(ethylene-co-butene) (PEB) and iPP/OBC (olefin block copolymer) [14,15,18]. For example, in iPP/OBC blends, the OBC is more mobile than iPP. Although OBC segments cannot crystallize with iPP, blending OBC with iPP can enhance the effective mobility of the crystallizable iPP, and in turn promotes the nucleation and crystallization in the blends. In weakly segregated PE/iPP oligomers, however, the slow iPP segments lead to less effective transport of PE segments to crystallizing PE nuclei, and result in a longer incubation time and a slower nucleus growth rate (Figure 6a).

**Figure 9:**
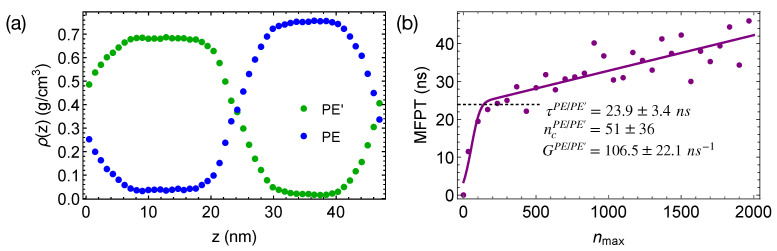
(**a**) Density profiles of phase-separated PE and PE’ oligomers at 420 K; (**b**) MFPT for the largest crystalline nucleus of PE to reach size $n_{max}$ in PE/PE’ blends. Fit to classical nucleation theory (curve).

To demonstrate the effect of polymer mobility on crystal nucleation in phase-separated blends, we construct a linear polymer of length 100 backbone atoms (PE’) that is incompatible with PE. The bonded interactions of PE’ are the same as those of TraPPE PE (Equations (2)–(4)). The intra-species non-bonded interactions of PE’ and the inter-species interactions between PE’ and PE are 0.8 and $0.8^{1/2}$ times of the VdW interactions of PE, respectively. The weaker VdW interactions of PE’ lead to a mismatch in cohesive energy density and hence the incompatibility between PE and PE’. A 50/50 blend of PE and PE’ phase separates at 420 K, creating wide interfaces and negligible interface-induced nematic order in the simulation box (Figure 9a). The weaker VdW interactions also lead to a lower crystal melting temperature and a higher mobility of PE’ than those of PE.

**Figure 10:**
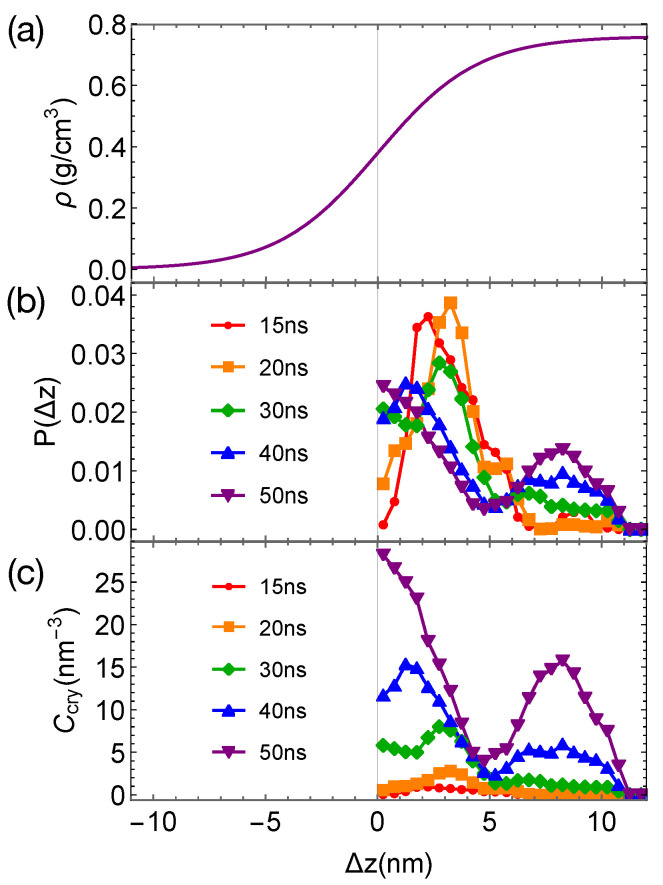
(**a**) Hyperbolic density profile of PE by fitting data in Figure 9a; (**b**) probability distribution of crystalline atoms vs. distance Δz from the closest planar interfaces at different simulation time; (**c**) concentration of crystalline atoms vs. Δz at different simulation times. Data in (**b**,**c**) are averaged over eight trajectories.

When the phase-separated blends of PE/PE’ are quenched to 300 K, crystal nucleation is only observed in the PE domains and the interfacial regions within 50 ns. The incubation time and the critical nucleus size are similar to those we observed in pure PE and PE${}_{a}$/iPP${}_{a}$ (Figure 9b). However, the growth of crystalline nuclei is somewhat faster in PE/PE’. We also observe that the crystalline atoms prefer to form in the interfacial region, where the concentration of PE is lower than the bulk value (Figure 10). In fact, PE prefers to nucleate in the interfacial regions where the volume fraction of PE is about 0.75, similar to the volume fraction of PE in the PE domains of the weakly segregated PE/iPP blends (see Figure 2). While 25% iPP oligomers impede the PE crystal nucleation and growth, the same amount of PE’ promotes PE crystallization. Similar to the role of OBC in the iPP/OBC blends [18], the more mobile PE’ segments enhance the dynamics of PE, and in turn promote the fast growth of PE nuclei in the interfacial regions.

The enhanced growth of crystalline nuclei in the interfacial region of weakly segregated PE/PE’ is different from that observed in strongly segregated PE${}_{a}$/iPP${}_{a}$. In strongly segregated PE${}_{a}$/iPP${}_{a}$, the inhomogeneous interfaces between the two incompatible domains are narrow. The growth of crystalline order occurs in the interface-induced alignment layers of PE${}_{a}$ in which the amount of iPP${}_{a}$ is negligible. In this case, the nematic order induced by the interfaces promotes the growth of crystalline order. In PE/PE’, however, the crystalline order of PE prefers to occur in the interfacial regions where about 25% PE’ are present and PE segments are disordered. The promoted growth of crystalline order near interfaces arises from the enhanced molecular mobility of PE segments, which is induced by the more mobile PE’.

## 5. Conclusions

Using MD simulations, we demonstrate that the crystal nucleation and growth in incompatible polymer blends under deep supercooling conditions are governed by the phase-separated blend morphologies and the dynamics of polymer segments. In weakly segregated polymer blends, a more mobile incompatible polymer in the interfacial regions can enhance the growth of the crystalline order of a crystallizable polymer. However, the incubation time remains the same as that in pure samples. On the other hand, small amounts of slower polymers in the domains of crystallizing polymers can significantly hinder the crystal nucleation and growth. By inducing anisotropic alignment near the interfaces of strongly segregated blends, phase separation can also promote the growth of crystalline order in the interfacial region. The surface-induced nematic order, however, only imposes a negligible effect on the incubation time and the formation of a critical nucleus.

## Figures and Tables

**Table 1:** Fitting parameters of MFPT for different polymer samples.

Polymer Sample	τ (ns)	$n_{c}$ (carbons)	*G* (carbons/ns)
Pure PE	25.5 ±3.2	61 ±32	83.5 ±7.9
PE/iPP	55.5±8.0	25 ±12	11.4 ±3.3
PE${}_{a}$/iPP${}_{a}$	33.7±4.4	62 ±33	66.2 ±10.6
PE/PE’	23.9±3.4	51 ±36	106.5 ±22.1

## Data Availability

Simulation input files and raw data are available from the corresponding author upon request.

## Appendix A

The crystalline atom distributions in highly incompatible PE${}_{a}$/iPP${}_{a}$ from four independent simulation trajectories are shown below:
